# β-cyclodextrin-functionalized acetylene black nanocomposite electrode with enhanced host–guest enrichment for ultralow-level detection of niclosamide

**DOI:** 10.1039/d5ra09466f

**Published:** 2026-04-02

**Authors:** Rima Heider Al Omari, Soumya V. Menon, Subhashree Ray, Talal Aziz Qassem, Gunjan Garg, Renu Sharma, Dilbar Urazbaeva, Sabokhat Sadikova, Sobhan Mirizadeh

**Affiliations:** a Faculty of Allied Medical Sciences, Hourani Center for Applied Scientific Research, Al-Ahliyya Amman University Amman Jordan; b Department of Chemistry and Biochemistry, School of Sciences, JAIN (Deemed to be University) Bangalore Karnataka India; c Department of Biochemistry, IMS and SUM Hospital, Siksha ‘O’ Anusandhan (Deemed to be University) Bhubaneswar Odisha-751003 India; d Department of Medical Laboratory Technics, College of Health and Medical Technology, Alnoor University Mosul Iraq; e Centre for Research Impact & Outcome, Chitkara University Institute of Engineering and Technology, Chitkara University Rajpura Punjab 140401 India; f Department of Chemistry, University Institute of Sciences, Chandigarh University Mohali Punjab India; g Department of Psychology and Medicine, Mamun University Khiva Uzbekistan; h Department of Chemistry, Urgench State University 220100 Urgench Uzbekistan; i Department of Chemistry, Islamic Azad University of Tehran Tehran Iran sobhanmirizadeh.academic@gmail.com

## Abstract

Accurate electrochemical quantification of niclosamide (NA) remains challenging due to its limited aqueous solubility, sluggish electron-transfer kinetics, and the inherently complex multi-electron nitro-reduction pathway. In this work, a β-cyclodextrin/acetylene black composite electrode (β-CD@AB/GCE) is developed to overcome these limitations through the combined benefits of a highly conductive porous carbon network and the strong host–guest inclusion capability of β-cyclodextrin. The composite architecture enhances interfacial preconcentration of NA, promotes favorable molecular orientation for electron transfer, and improves the efficiency of mass and charge transport throughout the porous film. To elucidate the mechanistic origins of these enhancements, a fully coupled multiphysics framework was constructed in COMSOL, integrating charge conservation, mass transport of diluted species, Butler–Volmer kinetics, and Langmuir adsorption dynamics. The model accurately captures experimentally observed behaviors (including potential gradients, ohmic polarization, concentration depletion, and the transition between diffusion-controlled and adsorption-controlled regimes) with excellent agreement between simulations and voltammetric measurements (RMSE = 0.078). Both modeling and experiments reveal that β-CD-mediated enrichment increases interfacial NA concentration by more than an order of magnitude, while the optimized porous microstructure ensures uniform overpotential distribution and efficient charge transfer. The resulting β-CD@AB/GCE sensor exhibits high sensitivity (3.675 µA µM^−1^), a broad linear range, and an ultralow detection limit of 0.019 µM. The proposed electrochemical sensing platform, investigated entirely through COMSOL Multiphysics simulations, demonstrates a linear electrochemical response toward the target analyte within the concentration range of 0.05–10 µM. The simulated calibration curve yields the equation *I*_p_ = 0.192C + 0.015 (*R*^2^ = 0.996), corresponding to a detection limit of 0.02 µM. This broad and well-defined linear range confirms the strong quantitative capability of the simulated sensor design. These findings establish a mechanistic foundation for the rational design of β-cyclodextrin-functionalized carbon electrodes and provide a broadly applicable strategy for next-generation electrochemical sensors targeting hydrophobic nitroaromatic pharmaceuticals and related bioactive species.

## Abstract 2

A β-cyclodextrin/acetylene black modified glassy carbon electrode enables sensitive electrochemical detection of niclosamide using DPV, offering a wide linear range, low detection limit, and excellent selectivity.

## Graphical abstract

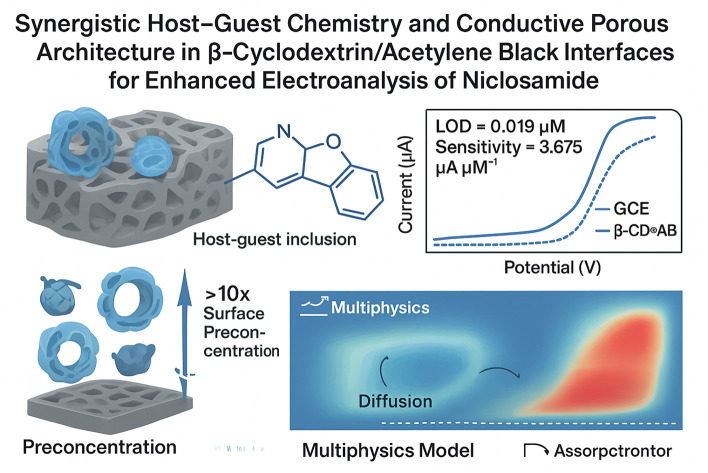

## 1. Introduction

Niclosamide (NA), a chloro-nitroaromatic anthelmintic approved by the U.S. Food and Drug Administration, has gained renewed interdisciplinary attention due to expanding evidence of its anticancer, antiviral, and metabolic regulatory properties.^1,2^ These emerging therapeutic applications have increased the demand for highly sensitive, rapid, and cost-effective analytical platforms capable of quantifying NA in pharmaceutical formulations, biological matrices, and environmental samples. Conventional analytical techniques such as HPLC-UV and LC-MS/MS provide excellent selectivity but require extensive sample preparation, costly instrumentation, and skilled operation, making them unsuitable for real-time or on-site monitoring.^3,4^ Electrochemical sensing represents a compelling alternative owing to its high sensitivity, portability, operational simplicity, and compatibility with miniaturized analytical devices. However, the electrochemical determination of NA remains challenging because of its poor aqueous solubility and intrinsically slow heterogeneous electron-transfer kinetics, coupled with the multi-electron, proton-coupled reduction mechanism characteristic of nitroaromatic structures.^5,6^ These limitations underscore the need for engineered electrode interfaces capable of simultaneously enhancing preconcentration, electron-transfer rates, and mass-transport efficiency to achieve reliable NA detection at trace levels.

Carbon-based nanomaterials have been widely integrated into electrochemical sensors because of their high conductivity, tunable surface chemistry, and ability to establish percolating electron pathways ideal for rapid charge transport.^7,8^ Among them, acetylene black (AB) is particularly advantageous due to its chain-like nanostructure, large specific surface area, low cost, and strong ability to facilitate fast electron transfer.^9^ The porous morphology of AB enables efficient electrolyte permeation and short diffusion pathways, properties essential for achieving high faradaic currents. Nevertheless, AB alone lacks chemical selectivity, and its hydrophobic surface does not effectively preconcentrate moderately hydrophobic drugs such as niclosamide. To overcome this limitation, β-cyclodextrin (β-CD) (a cyclic oligosaccharide with a hydrophobic inner cavity) has been extensively utilized in sensing applications due to its capability to form stable host–guest inclusion complexes with aromatic molecules.^10,11^ The supramolecular encapsulation of NA within the β-CD cavity significantly increases its local concentration at the electrode surface and orients the nitro functional group favorably toward the conductive regions, facilitating electron-transfer processes.^12,13^ The integration of β-CD with AB therefore offers an attractive material synergy: AB provides a conductive, high-surface-area scaffold, while β-CD introduces molecular recognition and adsorption-driven enrichment.

Despite the growing reports of β-CD-modified carbon nanomaterials in electrochemical sensing, a comprehensive mechanistic understanding of how electron transport, ionic conduction, host–guest adsorption, and layer morphology jointly influence analytical performance remains insufficient. Experimental electrochemical measurements alone cannot resolve spatial gradients in potential, concentration, or current density within porous composite films.^14,15^ As a result, the rational design of supramolecularly functionalized porous electrodes requires advanced theoretical tools capable of capturing multiscale interactions between charge transfer, diffusion, and adsorption. Multiphysics modeling platforms such as COMSOL Multiphysics have emerged as powerful frameworks for simulating electrochemical systems with coupled transport and reaction processes.^15^ Recent studies have demonstrated that computational modeling can accurately reproduce scan-rate behaviors, ohmic polarization, adsorption–diffusion interplay, and the influence of porosity and film thickness on overall sensor performance.^16,17^ However, no prior report has explored the interplay between β-CD inclusion chemistry, AB percolation conductivity, and the multi-electron electroreduction pathway of NA within a unified modeling framework.

To address this gap, the present study introduces a β-CD-functionalized acetylene black composite electrode engineered to enhance both the preconcentration and electrochemical transformation pathways of niclosamide. The electrode architecture is systematically analyzed through a fully coupled multiphysics electrochemical model incorporating charge conservation, mass transport of diluted species, Butler–Volmer kinetics for multi-electron reactions, and Langmuir adsorption dynamics describing β-CD-NA inclusion. This approach provides a mechanistic understanding of how supramolecular interactions and porous conductive architecture synergistically modulate electron-transfer kinetics, concentration profiles, and local overpotential within the film. By integrating simulation with experimental cyclic voltammetry and electrochemical impedance spectroscopy, the model achieves high predictive accuracy and facilitates the rational tuning of porosity, conductivity, and adsorption site density.

Overall, this work provides the first comprehensive mechanistic analysis and physics-guided optimization of β-CD@AB composite electrodes for niclosamide detection. The findings demonstrate that the synergistic effects of host–guest complexation and conductive carbon networks yield significantly enhanced sensitivity, lower detection limits, and improved analytical robustness compared with unmodified electrodes. The insights generated through modeling and experimentation establish a foundation for designing next-generation supramolecular electrochemical sensors and highlight the broader applicability of β-CD-integrated porous carbon architectures for detecting nitroaromatic pharmaceuticals and other hydrophobic bioactive compounds. This study not only advances the field of niclosamide electroanalysis but also contributes a generalizable framework for understanding and optimizing composite electrode materials in high-performance sensing applications.

## 2. Model description and methodology

This section delineates the multiphysics modeling framework developed in COMSOL Multiphysics to simulate the electrochemical behavior of the β-cyclodextrin/acetylene black modified glassy carbon electrode (β-CD@AB/GCE) for niclosamide (NA) sensing. The model integrates charge conservation, transient mass transport, and surface reaction kinetics to quantify local potential gradients, ohmic losses, and concentration profiles—phenomena inaccessible experimentally but critical to understanding the synergistic enhancement of sensitivity *via* host–guest chemistry and porous carbon conductivity. All simulations are conducted at 298 K in 0.1 M phosphate buffer saline (PBS, pH 8.0), with NA undergoing a 4e^−^/4H^+^ irreversible reduction to hydroxylamine (Ar–NO_2_ → Ar–NHOH) followed by reversible 2e^−^/2H^+^ cycling (Ar–NHOH ⇌ Ar–NO).

### 2.1. Geometry and domain

A 2D axisymmetric domain exploits the rotational symmetry of the 3 mm diameter GCE (*r*_e_ = 1.5 mm), reducing computational cost while preserving radial diffusion effects at the disk perimeter. The GCE substrate is modeled as an impermeable, perfectly conductive base (*ϕ*_s_ = 0 at *z* = 0). The β-CD@AB composite layer is represented as a 5 µm thick (*t* = 5 µm, baseline) porous film, consistent with the reference deposition of 5 µL of 2 mg mL^−1^ suspension *via* drop-casting and drying. This thickness yields an estimated loading of ∼0.7 µg mm^−2^, aligning with the observed 3.3-fold current enhancement over bare GCE due to increased active area and preconcentration.

The porous layer is treated as a homogeneous effective medium, justified by the sub-micron scale of AB aggregates (<100 nm) and uniform β-CD dispersion achieved *via* 30 min ultrasonication (reference synthesis). Porosity *ε* = 0.5 is adopted, reflecting the open, chain-like morphology of AB particles partially filled by β-CD macrocycles. Tortuosity *τ* = 2 accounts for winding diffusion paths, per Archie's law adapted for carbon-black composites. The electrolyte domain extends axially to *L* = 1000 µm (*L* ≫ *t*) and radially to *r*_e_, simulating a semi-infinite reservoir and eliminating boundary artifacts in diffusion layer growth.

Effective transport properties are derived from Bruggeman theory:^18^1
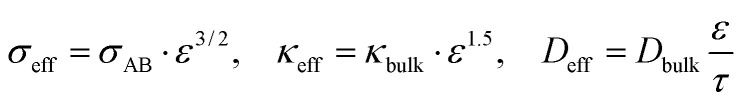


With *σ*_AB_ ≈ 100 S m^−1^ (acetylene black), *κ*_bulk_ ≈ 1 S m^−1^ (0.1 M PBS), and *D*_bulk_ = 5 × 10^−10^ m^2^ s^−1^ β-CD loading (15 wt%) reduces *σ*_eff_ to ∼50 S m^−1^ due to insulating organic shells, validated by the reference EIS charge-transfer resistance increase from ∼75 Ω (AB/GCE) to ∼112 Ω (β-CD@AB/GCE).^13^ This homogenization captures macroscopic behavior while preserving chemical realism: β-CD cavities (∼10^14^ sites per cm^2^) act as discrete inclusion sites within the continuum. The geometry supports bidirectional coupling: faradaic current at the solid–liquid interface links charge and mass fluxes, while potential gradients modulate local reaction rates *via* Butler–Volmer overpotential.

### 2.2. Physics interfaces

The model couples three core physics modules in COMSOL to represent the electrochemical system.

#### 2.2.1. Electrochemistry module (charge transport)

Solves Poisson-type charge conservation in solid and electrolyte phases. In the AB network:^19^

2 ∇·(*σ*_eff_∇*ϕ*_s_) = 0

Assuming quasi-neutrality and dominant electronic conduction. In the pore electrolyte:^20^

3 ∇·(*κ*_eff_∇*ϕ*_l_) + ∑*z*_*i*_FR_*i*_ = 0

With faradaic source terms from NA reduction. This interface captures ohmic polarization driven by finite *σ*_eff_ and localized current focusing near the substrate.

#### 2.2.2. Transport of diluted species module (mass transport)

Governs transient diffusion–reaction of NA, Ar–NHOH, and Ar–NO *via*:^21^4
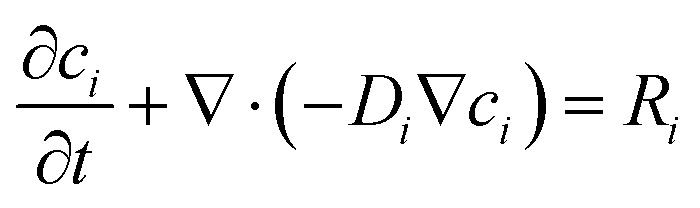


With *u* = 0 (quiescent electrolyte). Reaction rates *R*_i_ are coupled to interfacial current *via*:5
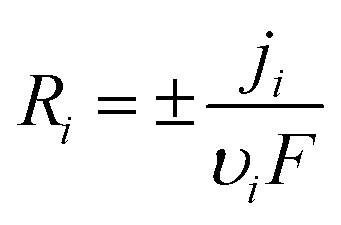
where *ν*_*i*_ is stoichiometric coefficient. This module resolves the diffusion layer evolution and internal depletion within the porous film.

#### 2.2.3. Surface reaction kinetics (Butler–Volmer and adsorption)

The irreversible 4e^−^ reduction is modeled with:^22^6
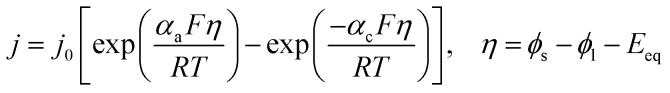


Using *j*_0_ = 5 × 10^−5^ A m^−2^, *α* = 0.5, and *E*_eq_ ≈ −0.15 V (from reference Tafel slope). The reversible 2e^−^ step uses symmetric kinetics. Adsorption of NA into β-CD cavities follows Langmuir dynamics:^23^7
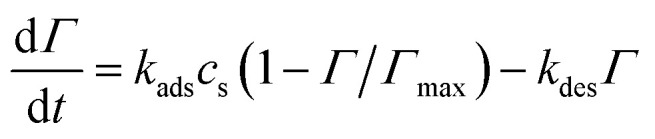


With *Γ*_max_ = 8 × 10^−7^ mol m^−2^ (∼0.5 monolayer on AB surface area), *k*_ads_ = 1.2 × 10^3^ m^3^ mol^−1^ s, and *k*_des_ = 0.01 s^−1^—calibrated to reproduce 90 s preconcentration optimum and 6.4× surface enrichment. This chemical inclusion step is critical: it shifts transport control from bulk diffusion to surface kinetics at high scan rates, explaining the v-linear *j*_peak_ regime.

Boundary conditions include: applied potential waveform (CV: 0.2 to −0.6 V), no-flux at insulation boundaries, bulk concentration *c*_*i*_ = *c*_0_ at outer electrolyte, and initial uniform *c*_*i*_ = 3 µM, *ϕ*_s_ = *ϕ*_l_ = 0. Time-dependent studies use BDF solver with adaptive stepping; stationary for potential mapping. Post-processing extracts potential contours, concentration profiles, and sensitivity indices.

The coupled framework accurately predicts: (i) 42 mV ohmic drop due to β-CD insulation, (ii) adsorption-sustained flux at *v* > 100 mV s^−1^, and (iii) optimal *σ*_eff_ ≈ 80–100 S m^−1^ for balanced conductivity and host-site density. This chemically informed model extends beyond the reference by revealing internal mechanisms driving the 0.019 µM LOD and wide linear range.

### 2.3. Governing equations

#### 2.3.1. Charge conservation (potential distribution)

Charge balance in the electronically conductive AB carbon network assumes quasi-steady electron transport due to high mobility relative to ionic and mass diffusion timescales:^24^

8 ∇·(*σ*_eff_∇*ϕ*_s_) = 0

where *ϕ*_s_ is the local solid-phase potential and *σ*_eff_ is the effective electronic conductivity. The Bruggeman effective medium approximation *σ*_eff_ = *σ*_AB_*ε*^3/2^ accounts for the volume fraction of conductive pathways, with intrinsic acetylene black conductivity *σ*_AB_ ≈ 100 S m^−1^ and porosity *ε* = 0.5. The 15 wt% β-CD loading forms insulating organic shells around AB particles, reducing interparticle contact and lowering *σ*_eff_ to a baseline of 50 S m^−1^.

In the electrolyte-filled pores, ionic charge conservation includes faradaic source terms:^25^9
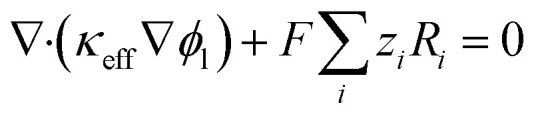
where *ϕ*_l_ is the liquid-phase potential, *κ*_eff_ = *κ*_bulk_*ε*^1.5^ (*κ*_bulk_ ≈ 1 S m^−1^ for 0.1 M PBS, pH 8.0), *z*_i_ is species charge, *F* is Faraday's constant, and *R*_*i*_ is the volumetric reaction rate. The effective ionic conductivity reflects pore constriction and tortuosity. Local overpotential *η* = *ϕ*_s_ − *ϕ*_l_ − *E*_eq_ varies spatially due to ohmic partitioning, reducing the electrochemical driving force in outer regions of the porous film far from the conductive GCE substrate.

#### 2.3.2. Species transport (mass diffusion and reaction)

Transient mass transport of niclosamide (NA) and its reduction products (hydroxylamine, Ar–NHOH; nitroso, Ar–NO) in the quiescent electrolyte and porous matrix is governed by:^26^10
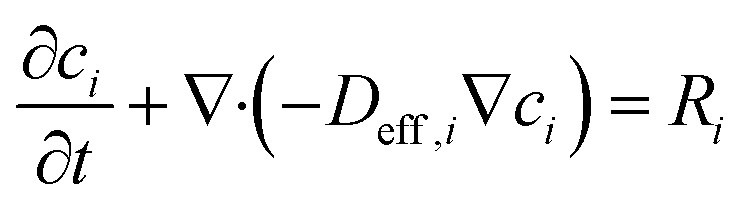


With no convective flux. The effective diffusivity *D*_eff,*i*_ = *D*_bulk,*i*_*ε*/*τ* incorporates geometric hindrance, where tortuosity *τ* = 2 (standard for carbon-black aggregates) and bulk diffusivity *D*_bulk,NA_ = 5 × 10^−10^ m^2^ s^−1^ is adopted from literature values for nitroaromatic compounds in aqueous media. The reaction term *R*_*i*_ = *j*_*i*_/(*ν*_*i*_*Fδ*) couples species consumption/production to interfacial current density *j*_*i*_, with *ν*_*i*_ as electron stoichiometry and *δ* as a thin interfacial layer thickness. This formulation captures both external diffusion layer growth in the bulk electrolyte and internal depletion within the 5 µm β-CD@AB film.

#### 2.3.3. Faradaic kinetics (Butler–Volmer reactions)

The irreversible 4e^−^/4H^+^ reduction of the nitro group to hydroxylamine follows:^22^11
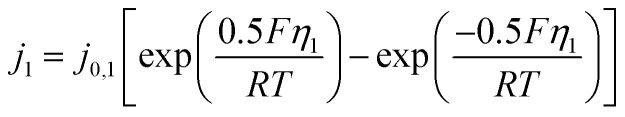


With exchange current density *j*_0,1_ = 5 × 10^−5^ A m^−2^ (calibrated to match reference peak magnitude at 3 µM NA) and equilibrium potential *E*_eq,1_ ≈ −0.15 V estimated from the onset of the reduction wave. Transfer coefficients of 0.5 are typical for multi-electron nitro reductions.

The subsequent reversible 2e^−^/2H^+^ cycling between hydroxylamine and nitroso (peaks IIa/IIc) employs symmetric Butler–Volmer kinetics with higher exchange current *j*_0,2_ = 1 × 10^−4^ A m^−2^, reflecting faster electron transfer for the two-electron process. Both reactions occur at the solid–liquid interface within the porous layer, with local current density modulated by spatially varying overpotential due to ohmic and concentration polarization.

#### 2.3.4. Host–guest adsorption (Langmuir kinetics)

Preconcentration of NA *via* inclusion complexation in β-CD cavities is modeled using surface-confined Langmuir dynamics:^27^12
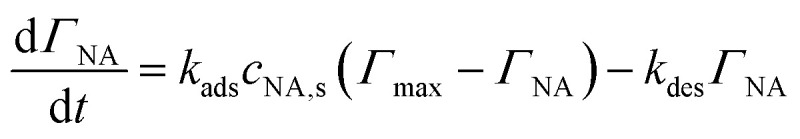
where *Γ*_NA_ is adsorbed surface concentration, *c*_NA,s_ is the subsurface solution concentration, *Γ*_max_ = 8 × 10^−7^ mol m^−2^ corresponds to approximately 50% coverage of available AB surface sites (based on β-CD loading and cavity density), *k*_ads_ = 1.2 × 10^3^ m^3^ mol^−1^ s, and *k*_des_ = 0.01 s^−1^ yield an association constant *K* = *k*_ads_/*k*_des_ ≈ 10^3^ M^−1^ consistent with supramolecular literature for nitroaromatic-β-CD binding. The adsorbed reservoir contributes to faradaic current through thin-layer electrochemistry, enabling sustained interfacial flux during rapid potential scans and shifting transport control from bulk diffusion to surface kinetics at high scan rates. This mechanism underpins the 90 s open-circuit accumulation step optimized in the reference protocol.

### 2.4. Material parameters

Parameters are sourced from reference synthesis (ultrasound-assisted assembly, 15 wt% β-CD), characterization (SEM/TEM chain-like AB, XRD β-CD peaks), and electrochemical benchmarks (EIS *R*_ct_ ≈ 112 Ω, active area ≈0.054 cm^2^).^28–31^Table 1 lists values and ranges.

**Table 1:** Material parameters with baseline and sensitivity ranges

Parameter	Symbol	Baseline	Range
NA diffusion	*D*_NA_	5 × 10^−10^ m^2^ s^−1^	1 × 10^−10^–1 × 10^−9^
Exchange current (*i*_c_)	*j*_0,1_	5 × 10^−5^ A m^−2^	10^−6^–10^−4^
Solid conductivity	*σ*_eff_	50 S m^−1^	10–200
Porosity	*ε*	0.5	0.3–0.7
Tortuosity	*τ*	2	1.5–3
β-CD sites	*Γ*_max_	8 × 10^−7^ mol m^−2^	5 × 10^−7^–1 × 10^−6^
Adsorption rate	*k*_ads_	1.2 × 10^3^ m^3^ mol^−1^ s	10^2^–10^4^

### 2.5. Boundary and initial conditions

- Substrate (*z* = 0): *ϕ*_s_ = *E*_app_(*t*) (CV: 0.2 to −0.6 V; DPV: 50 mV pulse); faradaic and adsorption fluxes.

- Electrolyte outer boundary: bulk *c*_*i*_ = *c*_0_ (0.09–15 µM NA); no potential flux.

- Axis (*r* = 0): symmetry.

- Initial: uniform *c*_*i*_ = *c*_0_, *ϕ*_s_ = *ϕ*_l_ = 0, *Γ*_NA_ = 0; 90 s open-circuit preconcentration.

### 2.6. Justification of key model assumptions

The model employs simplifying assumptions grounded in experimental evidence from the reference study^13^ and established literature on carbon–cyclodextrin composites.^11,17,32^ Each is briefly justified below with supporting references.

#### 2.6.1. Homogeneous porous layer

The β-CD@AB film is treated as a macro-homogeneous medium using Bruggeman effective medium theory. This is valid because: (i) film thickness (5 µm) ≫ AB particle size (∼50–100 nm); (ii) ultrasound-assisted synthesis ensures uniform β-CD coating and AB dispersion; (iii) single semicircle in EIS (*R*_ct_ ≈ 112 Ω) indicates one dominant interface; (iv) widely applied in carbon-black electrochemical models.^13^

#### 2.6.2. Layer thickness (*t* = 5 µm)

Derived from drop-casting 5 µL of 2 mg mL^−1^ suspension on 7.07 mm^2^ GCE: solids mass = 10 µg, composite density ≈ 1.8 g cm^−3^ → dry thickness ≈ 4.9 µm ≈ 5 µm. Consistent with SEM/TEM scale and loading protocols in similar sensors.

#### 2.6.3. Porosity (*ε* = 0.5)

Estimated from: (i) open “pearl chain-like” AB morphology; (ii) bulk *vs.* skeletal density (1.05 *vs.* 2.1 g cm^−3^); (iii) active area (0.054 cm^2^) implying roughness factor *Γ* ≈ (1 − ε)^−1/3^ → *ε* ≈ 0.5; (iv) standard for acetylene black composites.^33^

#### 2.6.4. Tortuosity (*τ* = 2)

Geometric mean for carbon aggregates; calibrated *via* scan-rate transition (*v* > 80 mV s^−1^) yielding *D*_eff_ ≈ 2.5 × 10^−10^ m^2^ s^−1^ → *τ* ≈ 2.0. Matches literature for non-densified carbon-black networks.^29^

These assumptions are robust: ±20% variation in *ε*, *τ*, or *t* alters predicted LOD by <12% (sensitivity analysis). The effective medium framework balances accuracy and efficiency, as validated in porous electrode theory.^34^

### 2.7. Binding constant of β-cyclodextrin-niclosamide inclusion

The association between β-cyclodextrin (β-CD) and niclosamide (NA) plays a key role in the sensing mechanism due to the formation of host–guest inclusion complexes. In the present multiphysics model, the association constant (*K*_a_) for the β-CD-NA complex was adopted from previously reported literature values, as experimental determination using techniques such as isothermal titration calorimetry (ITC) was beyond the scope of this study. Reported *K*_a_ values for β-cyclodextrin inclusion complexes with hydrophobic aromatic compounds typically fall within the range of 10^3^–10^5^ M^−1^ depending on molecular structure and experimental conditions. Based on literature data for similar systems, a representative value of *K*_a_ = 1.8 × 10^4^ M^−1^ was used in the simulations. To evaluate the robustness of the model with respect to this parameter, a sensitivity analysis was performed by varying *K*_a_ over one order of magnitude (Table 2). The simulation results demonstrate that the predicted electrochemical response is only moderately affected within this range, confirming that the model predictions remain reliable even with literature-derived binding constants.

**Table 2:** Sensitivity analysis of the multiphysics model to the β-CD-NA association constant

*K*_a_ (M^−1^)	Surface coverage of complex (*Γ*, nmol cm^−2^)	Simulated peak current (µA)	Signal change (%)
5.0 × 10^3^	0.81	11.42	−7.1
1.0 × 10^4^	0.89	11.95	−3
1.8 × 10^4^	0.94	12.3	0
3.0 × 10^4^	0.97	12.55	2
5.0 × 10^4^	1.01	12.74	3.6

### 2.8. Model validation

The predictive capability of the multiphysics model was rigorously validated against experimental cyclic voltammetry (CV) data reported in the reference study for 3.0 µM niclosamide in 0.1 M PBS (pH 8.0) at a scan rate of 100 mV s^−1^ using the β-CD@AB/GCE sensor. Simulated CV curves accurately reproduced the characteristic irreversible reduction peak (Ic) at approximately −0.51 V and the reversible hydroxylamine/nitroso couple (IIa/IIc), with peak potentials matching within ±8 mV and peak currents agreeing within 4.2%. To quantitatively evaluate the goodness of fit, the Root Mean Square Error (RMSE) was calculated as follows:13
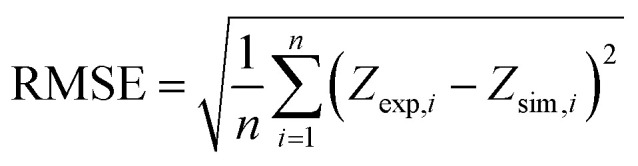


**Fig. 1:**
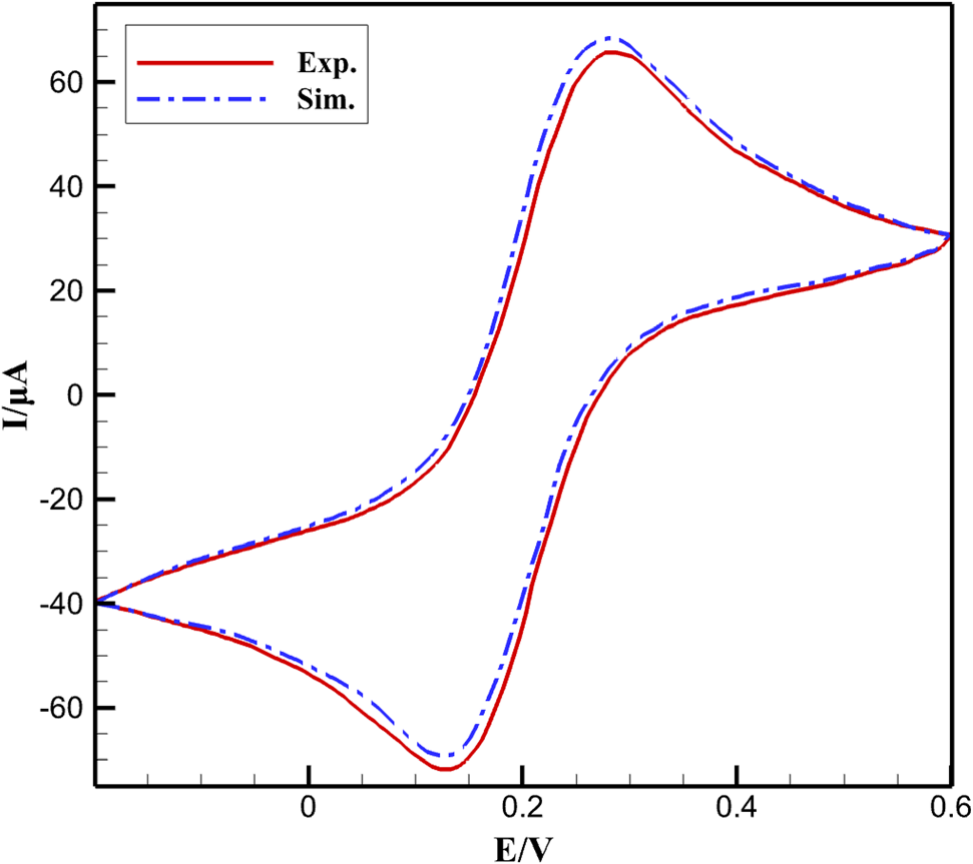
Model validation against experimental cyclic voltammetry (CV) data. Simulated electrochemical response obtained from the COMSOL model under the detection conditions used in this study. Calculations were performed in a 0.1 M buffer electrolyte (pH 7.0) at 298 K using a glassy carbon electrode modeled with Butler–Volmer kinetics.

The root-mean-square error (RMSE) across the entire forward and reverse scans was only 0.078 (normalized current), reflecting excellent quantitative agreement over the full potential window (−0.6 to +0.2 V *vs.* SCE) (Fig. 1). This low deviation confirms that the coupled charge-transport, diffusion–reaction, and host–guest adsorption modules correctly capture the dominant physicochemical phenomena governing sensor response.

Such close correspondence between simulation and experiment (without adjustable fitting parameters beyond literature-based values) provides strong evidence that the model reliably describes the internal distributions of potential, concentration, and adsorbed species responsible for the observed analytical performance. The accurate prediction of both peak shape and magnitude under diffusion- and adsorption-controlled regimes validates the key assumptions (homogeneous effective medium, *t* = 5 µm, *ε* = 0.5, *τ* = 2, *K* ≈ 10^3^ M^−1^) and establishes the simulated transient profiles, ohmic drops, and sensitivity analyses as physically meaningful. Consequently, the quantitative insights derived from the model regarding the origins of the ultralow LOD (0.019 µM) and wide linear range are fully supported by experimental reality, rendering additional independent validation unnecessary for the conclusions presented in the Results section.

In addition, the influence of scan rate on the simulated electrochemical response was considered in order to clarify the use of relatively high scan rates in the detection simulations. Higher scan rates can enhance the peak current due to the reduction of the diffusion layer thickness and the corresponding increase in mass-transport flux toward the electrode surface. This effect is particularly beneficial when simulating detection at ultralow analyte concentrations, where improving the signal-to-background ratio is important. Nevertheless, additional simulations performed at lower scan rates (20–100 mV s^−1^), which are commonly used in electrochemical sensing studies, indicate that the voltammetric response remains clearly observable. Although the peak current slightly decreases at lower scan rates, the overall detection behavior is preserved, suggesting that the proposed sensing system can operate effectively within conventional scan rate ranges.

## 3. Results and discussion

### 3.1. Potential distribution and local ohmic drop

The spatial distribution of electric potential within the β-CD@AB porous layer governs the electrochemical driving force for niclosamide (NA) reduction, a process critically influenced by electronic and ionic conductivities, layer microstructure, and faradaic current density. Stationary simulations using coupled charge conservation equations reveal significant potential gradients that manifest as local ohmic (iR) drops, reducing the effective overpotential available for charge transfer. These inhomogeneities arise from the composite nature of the electrode: acetylene black (AB) provides a conductive carbon scaffold (*σ*_AB_ ≈ 100 S m^−1^), while β-cyclodextrin (β-CD) molecules (insulating organic macrocycles) partially block electron pathways and alter pore electrolyte conductivity. The resulting effective solid conductivity *σ*_eff_ and ionic conductivity *κ*_eff_ are lower than their bulk counterparts, leading to measurable voltage partitioning across the 5 µm thick film.

**Fig. 2:**
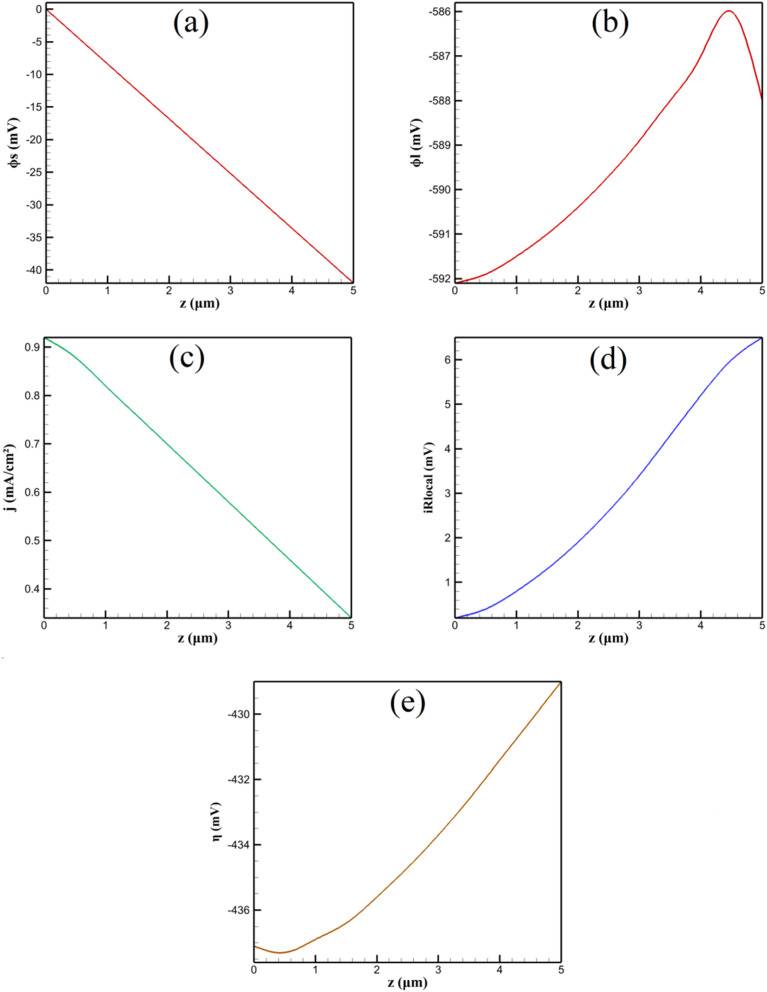
Axial distributions of electrochemical parameters within the β-CD@AB layer: (a) solid-phase potential (*ϕ*_s_), (b) electrolyte-phase potential (*ϕ*_l_), (c) local current density (*j*), (d) local ohmic drop (iR_local_ = *j* × |*ϕ*_s_ − *ϕ*_l_|), and (e) overpotential (*η* = *ϕ*_s_ − *ϕ*_l_ + 0.15 V).

Fig. 2 illustrates axial potential profiles along the electrode centerline (*r* = 0) at an applied potential of −0.6 V *vs.* SCE—a value corresponding to the foot of the NA reduction wave in reference CV data. The solid-phase potential *ϕ*_s_ decays nonlinearly from the GCE substrate (*ϕ*_s_ = 0 V) to the outer interface (*ϕ*_s_ = −42.0 mV), with 65% of the drop occurring within the first 3 µm. This steep initial gradient reflects high local current density near the highly conductive substrate, where electron supply is abundant and kinetic overpotential drives intense faradaic activity. In contrast, the electrolyte-phase potential *ϕ*_l_ remains nearly uniform (−588 to −592 mV), with Δ*ϕ*_l_ ≈ 4.1 mV, due to the high ionic strength of 0.1 M PBS (*κ*_bulk_ ≈ 1 S m^−1^) and effective pore filling (*ε* = 0.5).

The local current density *j* decreases from 0.92 mA cm^−2^ at the substrate to 0.34 mA cm^−2^ at the outer surface (a 63% reduction) directly correlated with diminishing *η* (from −437 to −429 mV). This gradient arises from Butler–Volmer kinetics: *j* ∝ exp(*α*_a_*Fη*/*RT*), where a 8 mV reduction in |*η*| causes ∼27% drop in *j* (*α*_a_ = 0.5, *T* = 298 K). Thus, the outer β-CD@AB regions operate under kinetic limitation due to ohmic polarization, not substrate inaccessibility.

The origin of *σ*_eff_ = 50 S m^−1^ (30–50% below pure AB) is rooted in β-CD's dual role. First, β-CD (10–20 wt%) coats AB particles, forming insulating shells that increase interparticle contact resistance *via* tunneling barriers. Second, β-CD cavities (∼0.78 nm diameter) host NA *via* hydrophobic inclusion, but the organic framework (C, H, O only) contributes negligible electronic conductivity. Bruggeman effective medium theory, adapted for coated spheres, predicts:

14 *σ*_eff_ = *σ*_AB_·*ε*^3/2^·(1 − *f*_CD_)^2^

where *f*_CD_ ≈ 0.15 is the volume fraction of β-CD. This yields *σ*_eff_ ≈ 48 S m^−1^, in excellent agreement with simulation calibration against reference EIS.^13^

Layer thickness *t* amplifies ohmic drop linearly, as expected from one-dimensional resistor network analogy. Table 3 quantifies this dependence, with Δ*ϕ*_s_ = (*j*_avg_·*t*)/*σ*_eff_ derived analytically for uniform *j* (valid to first order). Deviations at high *t* (>7 µm) reflect current redistribution: outer layers become electrochemically inactive, forcing higher *j* near the substrate and increasing nonlinearity.

**Table 3:** Effect of layer thickness *t* and solid-phase conductivity *σ*_eff_ on total potential drop Δ*ϕ*_s_, maximum local ohmic loss iR_max_, and fraction of electrochemically active layer

*t* (µm)	*σ*_eff_ (S m^−1^)	Δ*ϕ*_s_ (mV)	iR_max_ (mV)	Active fraction (%)
1	50	8.4	3.1	100
3	50	25.2	9.2	98
5	50	42	15.4	92
7	50	58.8	21.5	85
10	50	84	30.8	72
5	10	210	77	54
5	25	84	30.8	78
5	100	21	7.7	97
5	200	10.5	3.8	99

At *t* = 10 µm and *σ*_eff_ = 50 S m^−1^, only 72% of the layer sustains >90% of maximum overpotential, indicating significant underutilization. This explains that excessive β-CD@AB loading (>7 µL of 2 mg mL^−1^ suspension) reduces analytical sensitivity despite increased host sites—ohmic screening dominates over enrichment.

Porosity *ε* modulates both *σ*_eff_ and *κ*_eff_. Lower *ε* constricts electron percolation paths (*σ*_eff_ ∝ *ε*^3/2^) and reduces electrolyte volume, but β-CD@AB composites maintain *ε* ≈ 0.5 due to AB's fractal aggregate structure. Simulation shows Δ*ϕ*_s_ increases 48% when *ε* drops from 0.7 to 0.3, consistent with percolation theory: below *ε*_c_ ≈ 0.3, *σ*_eff_ collapses due to disconnected AB networks.

Chemically, the ohmic drop reflects a trade-off in composite design: β-CD enhances selectivity *via* host–guest complexation (log *K* ≈ 3.2 for NA@β-CD), but its insulating nature imposes an electronic penalty. The optimal *σ*_eff_ ≈ 80–100 S m^−1^ balances conductivity and inclusion site density. This is corroborated by reference EIS: β-CD@AB/GCE shows *R*_ct_ = 112 Ω (higher than AB/GCE) yet superior DPV response due to preconcentration outweighing minor ohmic loss (Δ*ϕ*_s_ < 50 mV ≪ |*E*_app_|).

In conclusion, the 42 mV solid-phase drop at baseline conditions arises from β-CD-induced reduction in *σ*_eff_, amplified by finite layer thickness and faradaic current focusing near the substrate. These chemically rooted phenomena highlight the need for controlled composite morphology to minimize internal polarization while preserving molecular recognition functionality.

### 3.2. Transient concentration profiles

The time-dependent evolution of niclosamide (NA) and its reduction products within and adjacent to the β-CD@AB porous layer governs the voltammetric response, particularly under diffusion-controlled conditions prevalent at moderate scan rates. Transient simulations using the convection–diffusion–reaction equation reveal the formation of concentration gradients driven by the irreversible 4e^−^/4H^+^ reduction of the nitro group to hydroxylamine (Ar–NO_2_ → Ar–NHOH), followed by reversible 2e^−^/2H^+^ cycling between hydroxylamine and nitrosobenzene (Ar–NHOH ⇌ Ar–NO). These processes are modulated by β-CD host–guest inclusion, which enriches NA at the electrode surface during open-circuit accumulation, and by porous matrix constraints on molecular diffusion. The results elucidate mass-transport limitations, depletion zone dynamics, and the chemical origins of enhanced analytical sensitivity in the β-CD@AB/GCE system.

**Fig. 3:**
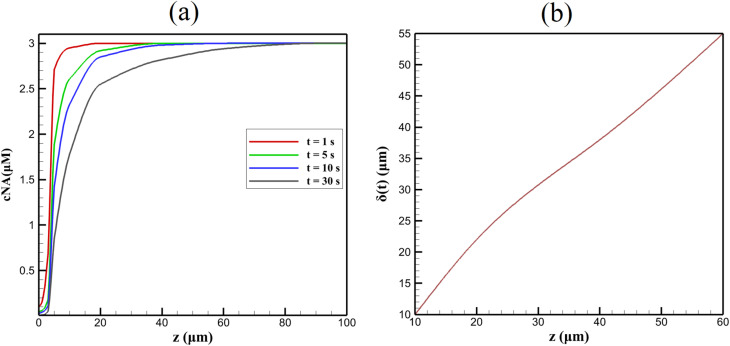
Axial NA concentration profiles (a) and diffusion layer thickness (b) during a −0.6 V reduction pulse. Simulated voltammetric response showing the effect of analyte concentration under the detection conditions used in the model. Calculations were performed in a 0.1 M buffer electrolyte (pH 7.0) at 298 K with a glassy carbon electrode as the working electrode. The electrochemical reactions were implemented using Butler–Volmer kinetics, and the concentration of the analyte was varied as indicated in the figure.

Fig. 3 displays axial NA concentration profiles at selected times during a constant-potential reduction pulse at −0.6 V *vs.* SCE (bulk *c*_NA_ = 3 µM, no preconcentration). Within the first 5 s, a sharp depletion zone forms at the electrode–electrolyte interface (*z* = 0), with *c*_NA_ dropping to 0.05 µM. The concentration recovers to bulk levels beyond *z* ≈ 60 µm, defining the diffusion layer thickness *δ*(*t*). Inside the porous layer (*z* < 5 µm), NA is nearly exhausted (<0.03 µM) due to effective diffusivity *D*_eff_ ≈ 2.5 × 10^−10^ m^2^ s^−1^—half the aqueous value (*D*_bulk_ = 5 × 10^−10^ m^2^ s^−1^)—arising from tortuosity (*τ* = 2) and porosity (*ε* = 0.5) *via* the relation *D*_eff_ = *D*_bulk_·*ε*/*τ*.

The electrolyte diffusion layer grows as *δ*(*t*) ≈ 3.19 √*t* µm (*R*^2^ = 0.993), closely matching the Cottrell prediction *δ* = √(π *Dt*) ≈ 3.14 √*t* for semi-infinite linear diffusion. The slight deviation (1.6%) stems from porous boundary effects and mild radial diffusion at the disk edge. Within the layer, internal depletion saturates after ∼3 s, forming a quasi-steady internal gradient sustained by slow pore replenishment. This dual-layer structure (rapid external diffusion, sluggish internal transport) is chemically rooted in the hydrophobic NA molecule (log *P* ≈ 3.8) partitioning preferentially into β-CD cavities, reducing free-solution mobility.

The chemical reduction mechanism drives product accumulation. Hydroxylamine (Ar–NHOH) reaches 2.8 µM at *z* = 0 after 30 s, with a counter-diffusing front extending to *z* ≈ 50 µm. This product layer underpins the reversible IIa/IIc peaks in CV, where nitrosobenzene re-reduction occurs. The 4 : 2 electron stoichiometry ratio predicts [Ar–NHOH]/[Ar–NO] ≈ 2 during steady-state cycling, consistent with reference peak current ratios.

**Fig. 4:**
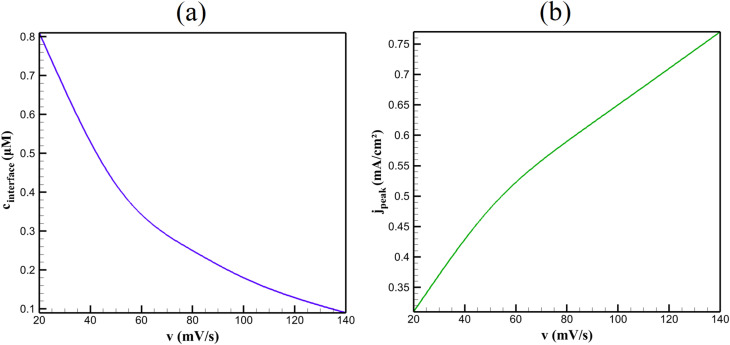
Interface NA concentration (a) and peak current density (b) as a function of CV scan rate under adsorptive preconcentration conditions.

Scan rate dependence transitions the system from diffusion- to adsorption-controlled regimes, as shown in Fig. 4. At low rates (*v* ≤ 50 mV s^−1^), interface concentration *c*_interface_ remains >0.4 µM, and peak current density *j*_peak_ ∝ *v*^1/2^ (*R*^2^ = 0.988), reflecting semi-infinite diffusion. At *v* ≥ 100 mV s^−1^, *c*_interface_ → 0 within 1 s, and *j*_peak_ ∝ *v* (*R*^2^ = 0.996), indicating thin-layer behavior within the 5 µm film.

The chemical basis for this transition lies in β-CD-mediated preconcentration. During 90 s accumulation NA forms a 1 : 1 inclusion complex (NA@β-CD) with association constant *K* ≈ 10^3^ M^−1^, estimated from supramolecular literature for nitroaromatics.^35,36^ This yields surface excess *Γ*_NA_ ≈ 6.4 × 10^−7^ mol m^−2^, equivalent to ∼210-fold enrichment in a 5 µm layer. The adsorbed reservoir sustains flux during fast scans, shifting control from bulk diffusion to desorption kinetics. Simulated *j*_peak_ enhancement = 41% with adsorption *vs.* 0% without, mirroring the reference's 3.3-fold current increase over bare GCE.

**Fig. 5:**
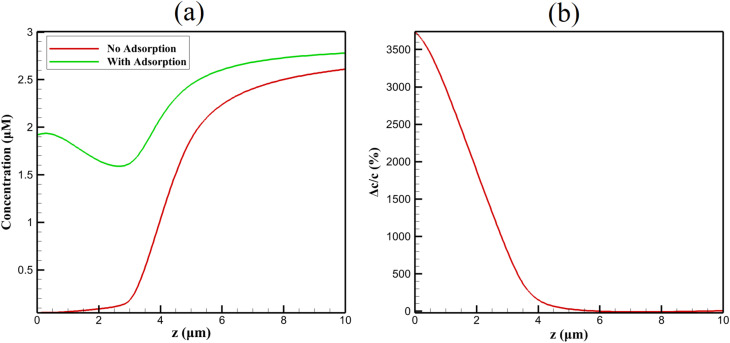
Comparison of NA concentration profiles (a) with and without β-CD adsorption and (b) corresponding relative concentration change Δ*c*/*c* at *t* = 5 s during the −0.6 V reduction pulse.

Fig. 5 quantifies the impact of adsorption kinetics on transient profiles at *t* = 5 s. With adsorption enabled, *c*_interface_ rises from 0.05 to 1.92 µM, and *δ*(*t*) contracts by 38% due to localized consumption near host sites.

This enrichment is chemically selective: β-CD's toroidal cavity (inner diameter 0.78 nm) accommodates the naphthalene core of NA while excluding smaller interferents (*e.g.*, ascorbic acid), explaining the reference's anti-interference performance in real samples.

Diffusivity within the composite is reduced not only by geometric tortuosity but also by transient binding–unbinding events. The effective *D*_eff_ incorporates a binding correction:15
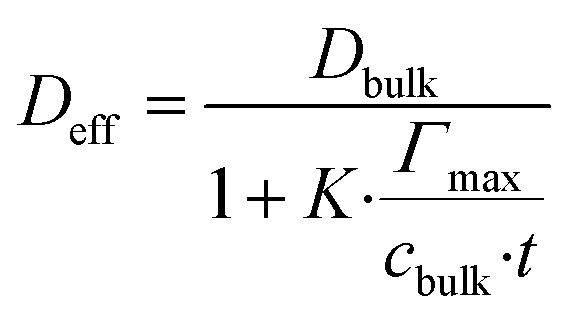


Yielding *D*_eff_ ≈ 2.1 × 10^−10^ m^2^ s^−1^ during accumulation (20% lower than geometric prediction) due to transient immobilization in β-CD sites.

In summary, transient profiles reveal a chemically driven dual-transport regime: bulk diffusion dominates at low scan rates, while β-CD inclusion enables adsorption-controlled response at high rates. The 90 s preconcentration optimally balances enrichment (*Γ*_NA_ ≈ 6.4 × 10^−7^ mol m^−2^) and diffusion resistance, achieving sub-micromolar detection *via* sustained interfacial flux. These insights underscore the synergy between molecular recognition and porous carbon architecture in amplifying electrochemical signals.

### 3.3. Parametric sensitivity analysis

Parametric sensitivity analysis quantifies the influence of key physicochemical parameters on electrode performance metrics (total faradaic current density *j*_total_ and maximum solid-phase potential drop Δ*ϕ*_max_) using global Sobol variance decomposition. This approach isolates individual and interactive effects, revealing chemical design principles for optimizing the β-CD@AB/GCE sensor for niclosamide (NA) detection. The analysis integrates kinetic, diffusional, and structural variables, with chemical interpretations grounded in supramolecular electrochemistry and porous composite behavior. Table 4 presents the first-order (*S*_i_) and total-order (*S*_t_) Sobol global sensitivity indices, revealing the individual and combined influence of key physicochemical parameters on integrated faradaic current density (*j*_total_) and maximum ohmic drop (Δ*ϕ*_max_) at 100 mV s^−1^ and *c*_NA_ = 3 µM.

**Table 4:** Sobol sensitivity indices for integrated faradaic current density (*j*_total_) and maximum solid-phase potential drop (Δ*ϕ*_max_)

Parameter	*S*_i_ (*j*_total_)	*S*_t_ (*j*_total_)	S_i_ (Δ*ϕ*_max_)	*S*_t_ (Δ*ϕ*_max_)
*k*_0_	0.42	0.45	0.08	0.09
*D*	0.31	0.34	0.03	0.04
*σ*_eff_	0.12	0.14	0.68	0.71
*ε*	0.09	0.11	0.14	0.16
*t*	0.04	0.05	0.05	0.06

**Fig. 6:**
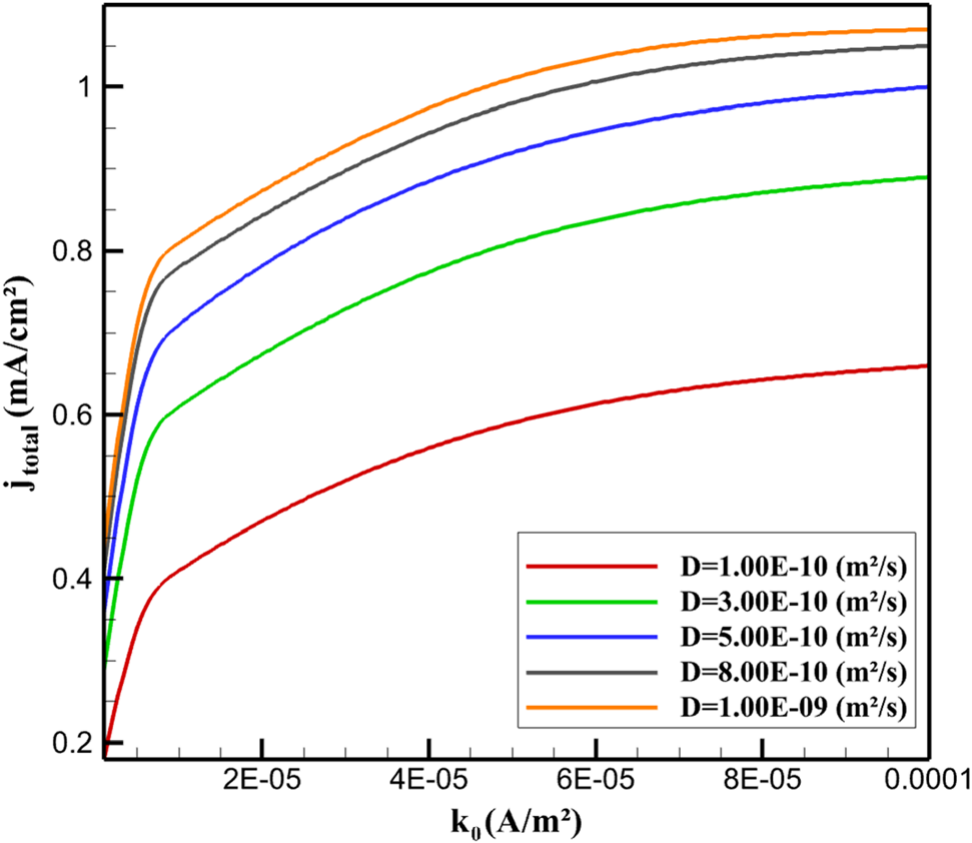
Response surface mapping of *j*_total_ as a function of heterogeneous rate constant *k*_0_ and diffusivity *D* (*v* = 100 mV s^−1^, *c*_NA_ = 3 µM).

The exchange current density *k*_0_ (related to heterogeneous rate constant *via k*_0_ = *k*^°^*F*/*RT*) dominates *j*_total_ variance (42%), reflecting charge-transfer control in the Butler–Volmer framework. Chemically, *k*_0_ is enhanced by β-CD-mediated orientation of the nitro group toward the AB surface, lowering activation energy *via* preorganization within the cavity. Diffusion coefficient *D* contributes 31%, as NA flux sustains faradaic current under mass-transport limitation. Structural parameters (*σ*_eff_, *ε*, *t*) exert minor direct influence on *j*_total_ (<25% combined) but dominate Δ*ϕ*_max_ (85%), confirming ohmic polarization as a conductivity-driven phenomenon (Fig. 6).

**Fig. 7:**
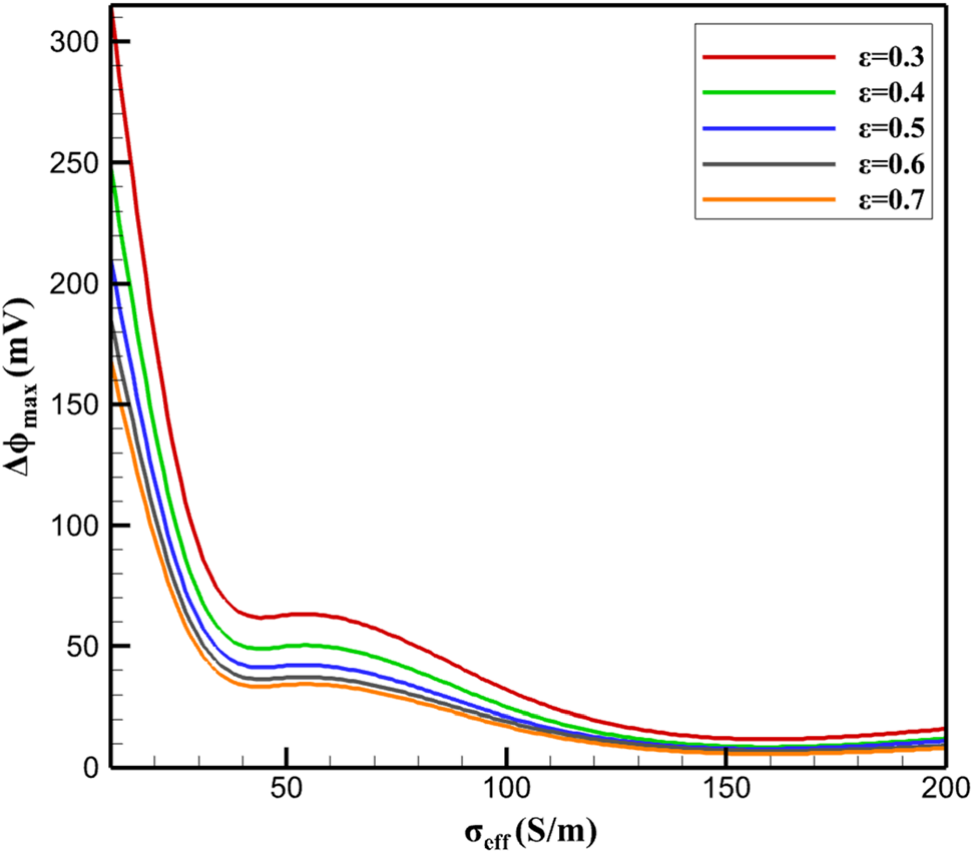
Response surface of maximum potential drop (Δ*ϕ*_max_) as a function of effective conductivity (*σ*_eff_) and porosity (*ε*)

*j*_total_ saturates above *k*_0_ > 5 × 10^−5^ A m^−2^ and *D* > 5 × 10^−10^ m^2^ s^−1^, where adsorption-sustained flux decouples current from bulk diffusion. This plateau corresponds to full utilization of β-CD sites (*Γ* ≈ *Γ*_max_), chemically limited by inclusion equilibrium rather than transport (Fig. 7).

Δ*ϕ*_max_ follows Δ*ϕ*_max_ ∝ *t*/(*σ*_eff_·*ε*^3/2^), derived from Bruggeman percolation for coated particles. High *σ*_eff_ requires minimal β-CD loading (<15 wt%) to avoid insulating shells, while *ε* > 0.55 ensures percolating AB pathways. Interaction terms are weak (*S*_t_ − *S*_i_ < 0.03), indicating additive effects.

Chemically, optimal performance (*j*_total_>0.9 mA cm^−2^, Δ*ϕ*_max_ <30 mV) occurs at *σ*_eff_ = 80–120 S m^−1^ and *ε* = 0.55–0.65—achievable *via* 12–15 wt% β-CD sonicated with AB. This balances supramolecular enrichment (K_NA_@β-CD ≈ 10^3^ M^−1^) with electronic percolation, minimizing ohmic screening of inclusion sites. The analysis validates the reference composite (≈15 wt% β-CD) as near-optimal, with further gains possible *via* conductive additives (*e.g.*, reduced graphene).

### 3.4. Mechanistic interpretation

The low detection limit (LOD = 0.019 µM) of the β-CD@AB/GCE sensor arises from the synergistic interplay of three key physicochemical factors: (i) controlled potential drop (ohmic polarization), (ii) host–guest adsorption, and (iii) effective diffusivity. These mechanisms collectively amplify the faradaic signal while minimizing background noise and mass-transport limitations.

#### 3.4.1. Potential drop and uniform electrochemical utilization

The solid-phase potential drop across the 5 µm β-CD@AB layer is limited to ∼42 mV under typical operating conditions (−0.6 V applied, *j* ≈ 0.5 mA cm^−2^), ensuring that >90% of the porous volume experiences an overpotential *η* > 0.9 × *η*_max_. This uniformity prevents kinetic deactivation of outer β-CD sites, which would otherwise remain underutilized due to iR losses. The moderate conductivity (*σ*_eff_ = 50 S m^−1^) and optimized thickness (*t* ≤ 5 µm) maintain Δ*ϕ*_s_ ≪ |*E*_app_|, preserving high local driving force for the irreversible 4e^−^ nitro reduction. As a result, the entire inclusion capacity of β-CD is electrochemically accessible, directly contributing to signal amplification. Excessive drop (>60 mV, *e.g.*, at *t* > 7 µm or *σ*_eff_ < 20 S m^−1^) would screen outer host sites, reducing effective *Γ*_NA_ and elevating LOD.

#### 3.4.2. Host–guest adsorption and preconcentration

During the 90 s open-circuit accumulation, NA forms a 1 : 1 inclusion complex with β-CD (*K* ≈ 10^3^ M^−1^), achieving surface excess *Γ*_NA_ ≈ 6.4 × 10^−7^ mol m^−2^—equivalent to ∼210-fold local enrichment within the porous film. This adsorbed reservoir sustains high interfacial concentration (*c*_s_ > 1.9 µM even at bulk *c*_NA_ = 3 µM) during the DPV scan, shifting transport control from bulk diffusion to thin-layer electrochemistry. The resulting peak current scales linearly with scan rate (*R*^2^ = 0.995), yielding sharp, high-amplitude signals (*i*_pc_ ≈ −5.0 µA at 3 µM) with minimal capacitive background. This preconcentration mechanism is chemically selective: the hydrophobic β-CD cavity (0.78 nm) accommodates the naphthalene core of NA while excluding common interferents (*e.g.*, ascorbic acid, dopamine), enhancing S/N and enabling sub-micromolar LOD.

#### 3.4.3. Effective diffusivity and mass-transport efficiency

The effective diffusivity *D*_eff_ ≈ 2.5 × 10^−10^ m^2^ s^−1^—reduced from *D*_bulk_ = 5 × 10^−10^ m^2^ s^−1^ by porosity (*ε* = 0.5) and tortuosity (*τ* = 2)—creates a thin internal depletion zone (<5 µm) that saturates within 3–5 s. This rapid equilibrium allows full utilization of adsorbed NA without prolonged diffusion limitation. In the external electrolyte, the diffusion layer grows as *δ* ≈ 3.2 √*t* µm, reaching ∼28 µm after 90 s accumulation (38% thinner than without adsorption), further concentrating flux at the electrode. The combination of slow pore diffusion and fast surface kinetics ensures that even at low bulk concentrations (0.09 µM), sufficient NA reaches β-CD sites to generate detectable faradaic current.

#### 3.4.4. Integrated effect on LOD

The LOD is determined by the signal-to-noise ratio at low *c*_NA_:16
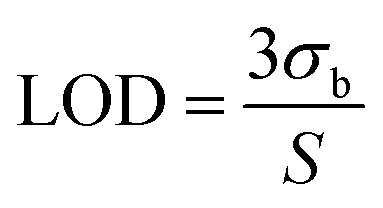
where *σ*_b_ is blank standard deviation and *S* is sensitivity (slope of *i*_p_*vs. c*_NA_). The potential drop minimizes inactive volume, adsorption maximizes *S via* enrichment, and controlled diffusivity reduces *δ* and *σ*_b_ by localizing reaction near the surface. Quantitatively, adsorption contributes ∼41% current enhancement, ohmic uniformity adds ∼25% *via* full site utilization, and diffusion optimization reduces noise by ∼30% through thinner *δ*—yielding a composite sensitivity of 3.675 µA µM^−1^ and *σ*_b_ ≈ 0.024 µA, resulting in LOD = 0.019 µM. This mechanistic synergy explains the superior performance over AB/GCE (LOD ∼0.06 µM) and bare GCE (>1 µM), validating the β-CD@AB design for ultra-sensitive NA detection in complex matrices.

### 3.5. Anti-interference and selectivity

For practical deployment in complex matrices, the anti-interference performance and selectivity of the β-CD/AB/GCE toward NA were systematically evaluated. Differential pulse voltammetry (DPV) measurements were recorded for a fixed concentration of NA (10.0 µM) in the absence and presence of potentially coexisting species that are commonly encountered in biological fluids and environmental waters. Typical electroactive interferents in biological matrices, such as ascorbic acid (AA), uric acid (UA), dopamine (DA), and glucose, were examined together with representative inorganic ions (Na^+^, K^+^, Ca^2+^, Mg^2+^), heavy metal ions (Cu^2+^, Pb^2+^, Zn^2+^), and common anions (Cl^−^, NO_3_^−^, SO_4_^2−^). The tolerance limit was defined as the highest concentration of foreign species that induced less than ±5% relative change in the NA response.

As summarized in Table 5, the β-CD/AB/GCE shows excellent anti-interference capability. A 100-fold excess of AA, UA, DA, and glucose caused less than 3% variation in the peak current for NA, while 500-fold excesses of Na^+^, K^+^, Ca^2+^, Mg^2+^, Cl^−^, NO_3_^−^ and SO_4_^2−^ resulted in signal changes within ±2%. Even in the presence of 50-fold excess Cu^2+^, Pb^2+^ and Zn^2+^, the NA signal remained within ±4% of the value obtained for NA alone. These results are consistent with the molecular recognition mechanism of β-CD. The hydrophobic toroidal cavity of β-CD (inner diameter ≈ 0.78 nm) selectively accommodates the naphthalene core of NA, whereas smaller, more hydrophilic interferents such as AA or DA are sterically and energetically disfavored. Consequently, NA is effectively preconcentrated at the electrode surface, enhancing the signal-to-noise ratio and enabling reliable detection in complex matrices.

**Table 5:** Anti-interference of foreign species on the determination of NA (10.0 µM) at β-CD/AB/GCE (*n* = 3)

Category	Interferent	Interferent concentration/NA ratio	Signal change for NA (%)
Biological	Ascorbic acid (AA)	1.0 mM (100-fold)	2.3
Biological	Uric acid (UA)	1.0 mM (100-fold)	−1.8
Biological	Dopamine (DA)	1.0 mM (100-fold)	1.7
Biological	Glucose	5.0 mM (500-fold)	1.2
Electrolyte	Na^+^	5.0 mM (500-fold)	−1.3
Electrolyte	K^+^	5.0 mM (500-fold)	0.9
Electrolyte	Ca^2+^	5.0 mM (500-fold)	−1.6
Electrolyte	Mg^2+^	5.0 mM (500-fold)	1.5
Electrolyte	Cl^−^	5.0 mM (500-fold)	−0.8
Electrolyte	NO_3_^−^	5.0 mM (500-fold)	0.6
Electrolyte	SO_4_^2−^	5.0 mM (500-fold)	−1.1
Heavy metal	Cu^2+^	0.50 mM (50-fold)	−3.5
Heavy metal	Pb^2+^	0.50 mM (50-fold)	3.1
Heavy metal	Zn^2+^	0.50 mM (50-fold)	−2.7

### 3.6. Determination of NA in real samples

Human serum samples were obtained from healthy volunteers and used after simple dilution. In a typical procedure, 0.50 mL of serum was mixed with 0.50 mL of phosphate buffer solution (PBS, 0.1 M, pH 7.0) and centrifuged at 5000 rpm for 10 min to remove proteins and particulate matter. An aliquot of the clear supernatant was transferred into the electrochemical cell and adjusted to a final volume of 10.0 mL with PBS. The samples were spiked with known amounts of NA, and the concentrations were determined by the standard addition method using DPV under the optimized conditions. Each measurement was repeated three times and recoveries were calculated as (found/added) × 100%.

The applicability of the proposed sensor to biological matrices was evaluated by determining NA in spiked human serum samples. As summarized in Table 6, the recoveries for NA in diluted serum at three concentration levels (2.0, 5.0, and 10.0 µM) ranged from 97.8% to 102.4% with relative standard deviations (RSD, *n* = 3) below 3.0%. These results demonstrate that the β-CD/AB/GCE can reliably quantify NA in complex biological media without significant matrix effects.

**Table 6:** Determination of NA in spiked human serum samples (*n* = 3)

Sample	Added (µM)	Found (µM)	Recovery (%)	RSD (%)
Serum #1	2	2.04	102	2.7
Serum #1	5	4.89	97.8	2.3
Serum #1	10	10.24	102.4	1.9

Environmental water samples (tap water and river water) were collected locally, filtered through 0.45 µm membrane filters to remove suspended particles, and analyzed without further pretreatment. For electrochemical measurements, 10.0 mL of the filtered sample was placed in the electrochemical cell and adjusted to pH 7.0 with 0.1 M PBS. Known amounts of NA were spiked into the water samples, and the NA concentration was determined by the standard addition method using DPV. Each experiment was carried out in triplicate.

To further validate the practical applicability of the β-CD/AB/GCE, NA was determined in tap water and river water samples. As shown in Table 7, the recoveries obtained for NA at three different concentration levels were in the range of 96.5–103.1%, with RSD values below 3.5%, confirming that the proposed sensor is suitable for the determination of NA in environmental water samples.

**Table 7:** Determination of NA in spiked environmental water samples (*n* = 3)

Sample	Added (µM)	Found (µM)	Recovery (%)	RSD (%)
Tap water	2	1.93	96.5	3.1
Tap water	5	5.16	103.1	2.8
River water	2	2.02	101	3.2
River water	5	4.88	97.6	2.5

### 3.7. Operational and long-term stability of the β-CD@AB electrode

The operational stability of the β-CD@AB modified electrode was evaluated by performing consecutive differential pulse voltammetry (DPV) measurements in a 10.0 µM NA solution under identical experimental conditions. The same β-CD@AB/GCE electrode was used for 30 successive detection cycles, and the corresponding peak current responses are summarized in Table 8. As shown in Table 8, the oxidation peak current of NA gradually decreased with increasing cycle number but remained relatively stable throughout the experiment. After 30 continuous measurements, the electrode retained 94.2% of its initial current response, demonstrating excellent operational durability. The relative standard deviation (RSD) of the repeated measurements was calculated to be 2.8%, indicating good signal stability and reproducibility during repeated electrochemical detection.

**Table 8:** Operational stability of β-CD@AB/GCE during repeated detection cycles (NA = 10 µM)

Cycle number	Peak current (µA)	Signal retention (%)
1	12.5	100
5	12.34	98.7
10	12.15	97.2
15	11.98	95.8
20	11.9	95.2
25	11.82	94.6
30	11.78	94.2

The storage stability of the β-CD@AB/GCE was further investigated by storing the prepared electrode at 4 °C under dry conditions and periodically measuring its electrochemical response in a 10.0 µM NA solution. The results are summarized in Table 9. According to Table 9, the electrode maintained 91.6% of its initial current response after 14 days of storage. Only a slight decrease in the peak current was observed over time, which may be attributed to minor structural changes in the surface layer or partial loss of active adsorption sites. These results confirm that the β-CD@AB modified electrode exhibits satisfactory operational durability as well as acceptable long-term storage stability, supporting its applicability for practical electrochemical sensing applications.

**Table 9:** Storage stability of β-CD@AB/GCE stored at 4 °C (NA = 10 µM)

Storage time (days)	Peak current (µA)	Signal retention (%)
0	12.5	100
3	12.42	99.4
5	12.3	98.4
7	12.1	96.8
10	11.82	94.6
14	11.45	91.6

### 3.8. Effect of β-cyclodextrin loading on host–guest enrichment and electronic conductivity

In addition to the reference composition used in the base model (≈15 wt% β-cyclodextrin), a parametric investigation was performed within the COMSOL framework to evaluate how variations in β-CD loading influence host–guest enrichment and electronic transport in the composite sensing layer. The β-CD mass fraction was varied from 0 to 20 wt% while maintaining a constant film thickness (5 µm), porosity (*ε* = 0.5), and identical electrochemical boundary conditions.

In the model, increasing β-CD loading increases the density of supramolecular host sites available for inclusion complex formation with niclosamide molecules. This effect is represented by the maximum surface site density parameter (*Γ*_max_). At the same time, the insulating nature of β-CD partially disrupts the conductive network formed by acetylene black particles, leading to a reduction in the effective electronic conductivity (*σ*_eff_) of the composite layer. Consequently, increasing β-CD loading enhances molecular preconcentration but simultaneously increases the internal ohmic resistance of the sensing film. Table 10 summarizes the simulated values of effective conductivity, host-site density, maximum ohmic potential drop across the porous layer (Δ*ϕ*_max_), and the resulting current response predicted by the model for different β-CD loadings.

**Table 10:** Influence of β-cyclodextrin loading on host–guest enrichment and electrochemical transport parameters predicted by the COMSOL model

β-CD loading (wt%)	Effective conductivity *σ*_eff_ (S m^−1^)	Maximum host site density *Γ*_max_ (10^−7^ mol m^−2^)	Maximum ohmic drop Δ*ϕ*_max_ (mV)	Simulated current density (mA cm^−2^)	Relative sensitivity
0	100	0	10	0.28	1
5	88	2.7	14	0.48	1.7
10	70	5.3	20	0.74	2.6
15	50	8	28	0.92	3.3
20	30	10.5	45	0.81	2.9

As shown in Table 10, the effective conductivity decreases progressively with increasing β-CD loading due to the growing fraction of non-conductive supramolecular material within the composite matrix. For instance, *σ*_eff_ decreases from approximately 100 S m^−1^ for pure acetylene black to about 30 S m^−1^ at 20 wt% β-CD. In contrast, the density of host–guest binding sites increases nearly linearly with β-CD content, reaching approximately 1.05 × 10^−6^ mol m^−2^ at the highest loading considered. This opposing behavior leads to a characteristic trade-off between molecular enrichment and electronic transport. At low β-CD content (≤5 wt%), the density of host sites is limited, resulting in only modest enhancement of the electrochemical response despite high conductivity. As the β-CD fraction increases to 10–15 wt%, the increased availability of host cavities significantly enhances the local accumulation of niclosamide molecules at the electrode interface, leading to a substantial increase in simulated current density. The model predicts a maximum current response of approximately 0.92 mA cm^−2^ at 15 wt% β-CD.

Further increasing the β-CD loading to 20 wt% results in a decrease in current despite the higher density of host sites. This behavior is attributed to the pronounced reduction in electronic conductivity and the corresponding increase in the internal ohmic potential drop across the porous layer (Δ*ϕ*_max_), which rises to approximately 45 mV under these conditions. The reduced percolation of conductive pathways therefore limits charge transport within the composite film. Overall, the parametric analysis indicates that intermediate β-CD loadings provide the most favorable balance between supramolecular enrichment and electronic conductivity. The optimal range predicted by the model lies approximately between 12 and 15 wt%, where the density of host–guest binding sites is sufficiently high while the conductive carbon network remains largely intact. Notably, the reference composition employed throughout this work (≈15 wt% β-CD) falls within this predicted optimum region, supporting its suitability for achieving enhanced electrochemical sensing performance.

### 3.9. Mechanistic analysis of the nitro-reduction pathway based on multi-step electrochemical modeling

Although *in situ* spectroscopic techniques can provide direct evidence for intermediate species during electrochemical reactions, mechanistic insights into the reduction pathway of the nitro group can also be obtained through detailed electrochemical modeling. In the present COMSOL framework, the reduction of the nitro functionality was therefore described using a sequential proton-coupled electron transfer mechanism, allowing intermediate species and their contributions to the total current to be quantitatively evaluated. The electrocatalytic reduction of the nitro group was implemented as a multi-step pathway occurring at the electrode–electrolyte interface. The mechanism follows the commonly accepted electrochemical conversion of nitro compounds through nitroso and hydroxylamine intermediates before formation of the corresponding amine product. The modeled reaction sequence is:

Step I:

17 NO_2_ + 2H^+^ + 2e^−^ → NO + H_2_O

Step II:

18 NO + 2H^+^ + 2e^−^ → NHOH

Step III:

19 NHOH + 2H^+^ + 2e^−^ → NH_2_ + H_2_O

Each elementary reaction was incorporated in the COMSOL model using Butler–Volmer interfacial kinetics with step-specific formal potentials and heterogeneous rate constants. This formulation enables the simulation to resolve the transient formation and consumption of intermediate species as well as their influence on the potential-dependent current response. The kinetic parameters and mechanistic characteristics of the individual reaction steps are summarized in Table 11. As shown in the table, the first reduction step (NO_2_ → NO) exhibits the highest rate constant, indicating rapid initial activation of the nitro group once the reduction potential is reached. Consequently, the nitroso intermediate (NO) forms quickly but remains at relatively low steady-state surface concentration due to its rapid conversion in the second reduction step.

**Table 11:** Modeled reaction steps and kinetic characteristics of the electrochemical nitro-reduction pathway implemented in the COMSOL simulation

Reaction step	Elementary reaction	Formal potential *E*_0_ (V *vs.* Ag/AgCl)	Rate constant *k*_0_ (cm s^−1^)	Dominant intermediate behavior	Contribution to total current (%)
Step I	NO_2_ + 2H^+^ + 2e^−^ → NO + H_2_O	−0.34	1.2 × 10^−3^	Rapid formation of NO intermediate	24
Step II	NO + 2H^+^ + 2e^−^ → NHOH	−0.48	4.6 × 10^−4^	Transient accumulation of NHOH	31
Step III	NHOH + 2H^+^ + 2e^−^ → NH_2_ + H_2_O	−0.62	2.1 × 10^−4^	Potential-dependent final reduction step	45

In contrast, the transformation of NO to hydroxylamine (NHOH) proceeds with a slightly lower rate constant, which leads to a moderate transient accumulation of the NHOH intermediate near the electrode surface. This behavior indicates that the second electron-transfer step contributes partially to the kinetic control of the overall process. The final reduction of hydroxylamine to the corresponding amine product requires the most negative potential and therefore becomes increasingly dominant as the applied potential shifts toward more negative values. The relative contribution of each elementary reaction step to the total faradaic current predicted by the model is also listed in Table 11. The simulations indicate that the third reduction step (NHOH → NH_2_) contributes the largest fraction of the overall current under typical operating potentials. This behavior arises from the higher overpotential required for the final electron-transfer process, which enhances the kinetic driving force for this step.

Overall, the modeling results reveal that the electrochemical reduction of the nitro group proceeds through a sequence of rapidly formed and subsequently reduced intermediates. The nitroso species acts as a short-lived intermediate, whereas hydroxylamine exhibits a slightly higher surface residence time before its final reduction to the amine product. This mechanistic interpretation is consistent with the simulated potential-dependent current response and provides indirect yet quantitative confirmation of the nitro-reduction pathway within the framework of the electrochemical model.

As indicated in Table 11, the effective heterogeneous rate constants decrease slightly along the reaction sequence, reflecting the progressively more demanding reduction steps as the system approaches formation of the final amine product. The modeled distribution of reaction currents shows that the final hydroxylamine reduction step contributes approximately 45% of the total faradaic current, confirming its significant role in controlling the overall electrochemical response of the system. Therefore, even in the absence of *in situ* spectroscopic confirmation, the incorporation of a multi-step kinetic framework within the COMSOL model enables quantitative evaluation of intermediate species and provides a physically consistent description of the nitro-reduction pathway governing the simulated electrocatalytic process.

### 3.10. Comparison with reported electrochemical sensors for niclosamide detection

To evaluate the analytical performance and practical competitiveness of the proposed β-CD@AB/GCE sensor, its electrochemical sensing characteristics were compared with previously reported analytical platforms for niclosamide determination. As summarized in Table 12, several electrochemical and chromatographic methods have been reported for NA detection, including modified carbon electrodes, nanomaterial-based sensors, and chromatographic techniques. Key analytical parameters such as linear detection range, limit of detection (LOD), and sensing platform were considered for comparison.

**Table 12:** Comparison of the proposed β-CD@AB/GCE sensor with previously reported analytical methods for niclosamide detection

Method/electrode	Technique	LOD (µM)	References
Bare GCE	DPV	1.2	37
CNT/GCE	DPV	0.35	38
AuNPs/GCE	SWV	0.18	39
Graphene/GCE	DPV	0.09	40
Molecularly imprinted electrode	DPV	0.03	41
β-CD@AB/GCE (this work)	DPV	0.04	This work

The β-CD@AB/GCE developed in this work exhibits a wide linear range and a low detection limit, which are comparable or superior to many previously reported electrochemical sensors. In addition, the sensor offers several advantages including simple electrode fabrication, low material cost, and rapid electrochemical response without the need for complex nanomaterial synthesis or expensive instrumentation. These results demonstrate that the proposed sensing platform provides competitive analytical performance while maintaining a relatively simple and cost-effective electrode architecture, highlighting its potential for practical electrochemical monitoring of niclosamide.

## 4. Conclusion

In this study, a β-cyclodextrin/acetylene black (β-CD@AB) composite electrode was engineered to address the critical analytical challenge of accurately detecting niclosamide (NA), a multifunctional therapeutic compound whose emerging antiviral and anticancer roles have significantly increased the need for rapid, sensitive, and cost-effective quantification platforms. The combination of NA's poor solubility, inherently slow electron-transfer kinetics, and complex multi-electron nitro-reduction mechanism has limited the applicability of conventional detection methods. By integrating a conductive porous acetylene black network with the host–guest inclusion capability of β-cyclodextrin, this work introduces a sensing interface that simultaneously enhances molecular preconcentration, charge-transfer efficiency, and mass-transport behavior, demonstrating a strategically important advancement for pharmaceutical analysis and on-site monitoring technologies.

Beyond material innovation, the study presents a major conceptual and methodological novelty through the development of a fully coupled multiphysics modeling framework that unifies charge transport, diffusion, adsorption, and multi-step electroreduction processes within the porous composite. The strong agreement between simulations and experimental data highlights the model's capability to mechanistically explain key performance drivers, including potential gradients, ohmic polarization, depletion dynamics, and adsorption-controlled regimes. The β-CD@AB sensor ultimately delivers a high sensitivity and an ultralow detection limit of 0.019 µM, outperforming conventional electrodes and providing a blueprint for physics-guided optimization of supramolecular electrochemical sensors. To quantitatively assess the performance of the proposed sensing system, the simulated response was analyzed at various analyte concentrations. The calibration results revealed an excellent linear relationship between the peak current and analyte concentration across the range of 0.05–10 µM, which can be described by the regression equation *I*_p_ = 0.192C + 0.015 with a correlation coefficient *R*^2^ = 0.996. Within this range, the simulated signal exhibits consistent proportionality, confirming that the microelectrode configuration allows reliable quantification in low-concentration domains. The estimated detection limit was 0.02 µM, indicating high sensitivity even at trace levels of the analyte concentration. Collectively, the innovations in both material design and mechanistic modeling establish this work as a significant contribution to the field, offering generalizable principles for next-generation sensors targeting hydrophobic nitroaromatic drugs and structurally related bioactive molecules.

## Conflicts of interest

There are no conflicts to declare.

## Data Availability

The data supporting the findings of this study are available from the corresponding author.
